# Cell competition from development to neurodegeneration

**DOI:** 10.1242/dmm.048926

**Published:** 2021-06-30

**Authors:** Carolina Costa-Rodrigues, Joana Couceiro, Eduardo Moreno

**Affiliations:** Champalimaud Centre for the Unknown, 1400-038 Lisbon, Portugal

**Keywords:** Alzheimer's disease, Fitness fingerprints, *azot* gene, Selective neuronal vulnerability, Supercompetition, *Drosophila melanogaster*

## Abstract

Cell competition is a process by which suboptimal cells are eliminated to the benefit of cells with higher fitness. It is a surveillance mechanism that senses differences in the fitness status by several modes, such as expression of fitness fingerprints, survival factor uptake rate and resistance to mechanical stress. Fitness fingerprints-mediated cell competition recognizes isoforms of the transmembrane protein Flower, and translates the relative fitness of cells into distinct fates through the Flower code.

Impairments in cell competition potentiate the development of diseases like cancer and ageing-related pathologies. In cancer, malignant cells acquire a supercompetitor behaviour, killing the neighbouring cells and overtaking the tissue, thus avoiding elimination. Neurodegenerative disorders affect millions of people and are characterized by cognitive decline and locomotor deficits. Alzheimer's disease is the most common form of dementia, and one of the largely studied diseases. However, the cellular processes taking place remain unclear.

*Drosophila melanogaster* is an emerging neurodegeneration model due to its versatility as a tool for genetic studies. Research in a *Drosophila* Alzheimer's disease model detected fitness markers in the suboptimal and hyperactive neurons, thus establishing a link between cell competition and Alzheimer's disease.

In this Review, we overview cell competition and the new insights related to neurodegenerative disorders, and discuss how research in the field might contribute to the development of new therapeutic targets for these diseases.

## Abstract (precis)

**Summary:** This Review presents cell competition as an active selection mechanism, from its more established molecular pathways to the recently proposed ones in neurodegenerative diseases.

## Introduction

Organ and tissue homeostasis is maintained by a balance between cell division and cell death. In disease states, a disturbance in this delicate balance leads to a general decline in physiological organ function and eventual death. In the nervous system, periods of cell division and proliferation occur in earlier stages of development and later, during adult neurogenesis from neural stem cells (Hollville et al., 2019; Wang et al., 2011). Once a mature and functional nervous system is established, expendable neurons undergo apoptosis (see Glossary, Box 1), and neuronal sensitivity to apoptosis decreases to ensure that the healthiest and fittest neurons survive (Hollville et al., 2019). In the brain, neuronal fate is also adjusted to increase the efficiency of neural circuits. The aberrant regulation of cell death mechanisms, and the increased neuronal vulnerability caused by an accumulation of errors throughout ageing, leads to the progression of several neurodegenerative diseases (NDDs), such as Alzheimer's disease (AD) (Chauhan et al., 2020; Mattson, 2000). NDDs are devastating illnesses and predominantly affect elderly people (Hirth, 2012). Memory deficits, cognitive impairment, decreased movement control and loss of sensation/touch are some of the NDD symptoms (Ambegaokar et al., 2010). NDDs are characterized by pathological protein misfolding and aggregation, impairments in their clearance, an increase in reactive oxygen species (ROS) levels, DNA damage, mitochondrial dysfunction, endoplasmic reticulum stress, and, ultimately, synaptic loss and neuronal death (Chi et al., 2018). Neurons are post-mitotic cells and cannot be directly replaced, so the earlier neurodegeneration is detected, the more therapeutics can be implemented to delay the progression of NDDs. Understanding the mechanisms behind neurodegeneration can bring new tools to the fight against these disorders. The sequence of events responsible for neurodegeneration is still controversial; numerous molecular mechanisms and receptors are potentially involved, and different, or even the same, mechanism can exert multiple deleterious effects, depending on the progression of the disease (Benilova et al., 2012). Heritable forms of these proteinopathies are associated with genetic defects, suggesting that the affected protein is causally related to the disease aetiology and/or progression (Bertram and Tanzi, 2005). However, human genetic studies are limited, making it necessary to use model systems to analyse affected genes and pathways in detail.

There is a high degree of genomic conservation between *Drosophila melanogaster* and *Homo sapiens*, which means that fundamental cellular processes, such as gene expression and regulation, membrane trafficking, neuronal connectivity and synaptogenesis, cell signalling and cell death, are often conserved (Ambegaokar et al., 2010).

*Drosophila* is a well-characterized invertebrate and often-used model for genetic manipulation due to its vast genetic toolkit, ease of use and fast data acquisition (McGurk et al., 2015). Because ∼75% of human disease-causing genes have functional homologues in flies, *Drosophila* has been recognized as a valuable model system in the study of human diseases (Aryal and Lee, 2019; Bilen and Bonini, 2005; Bolus et al., 2020; Marsh and Thompson, 2006; Pandey and Nichols, 2011; Prüßing et al., 2013). These studies describe reliable modelling of AD, Parkinson's disease (PD) and motor neuron diseases, as well as trinucleotide repeat expansion diseases like Huntington's disease (HD). The fruit fly can also be used to screen chemical compounds for their potential to prevent or ameliorate symptoms (Lenz et al., 2013; Qurashi et al., 2012; Rimkus and Wassarman, 2018; Ugur et al., 2016), which in turn can be a starting point for clinical research and the development of novel therapeutic strategies for the treatment of human NDDs.

Santiago Ramon y Cajal proposed in 1894 that developing neurons may be engaged in a competitive struggle for space and nutrition. This idea was confirmed by the neurotrophic theory and the discovery of nerve growth factor by Rita Levi-Montalcini (Levi-Montalcini, 1987). More recently, the somatic mutation theory of ageing proposed that although some impaired cells die, other damaged-but-still-viable and thus suboptimal ones remain in tissues and affect the homeostasis of the organism (Kennedy et al., 2012; Moskalev et al., 2013; Szilard, 1959). As a result, less-fit cells (Box 1) accumulate throughout life and may be eliminated from the organism by various mechanisms. One such mechanism is a conserved process called cell competition, which was first described in *Drosophila* in 1975 by Ginés Morata and Pedro Ripoll (Morata and Ripoll, 1975) and has recently been reported to occur in vertebrates as well (Eisenhoffer et al., 2012; Madan et al., 2019; Petrova et al., 2012). In cell competition (Box 1), suboptimal cells, called loser cells (Box 1), are eliminated from the tissue when confronted with more-optimal cells in their vicinity, called winner cells (Box 1), to maintain the homeostasis.

**Fig. 1.**
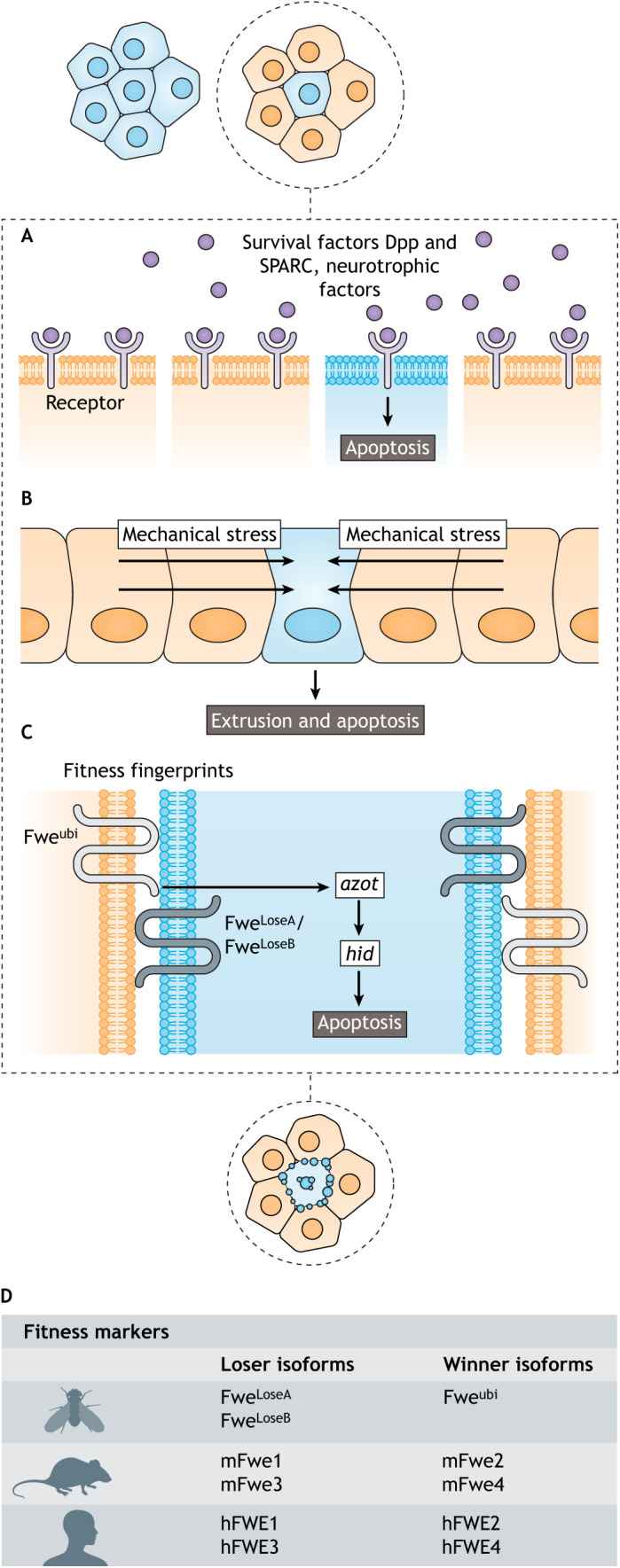
**Different modes of cell competition.** (A) Competition for limiting survival factors. Winner cells (orange) have a higher capacity to bind pro-survival factors, reducing the abundance of available survival factors for loser cells (blue). Note that loser cell death can occur without direct contact with winner cells. (B) Mechanical cell competition. Differential growth and/or tissue movement leads to the compression of loser cells (arrows), which triggers their elimination. Note that loser cell death does not require direct contact with winner cells. (C) Competition through comparison of fitness fingerprints. Loser cells express a less-fit Flower protein (Fwe^LoseA^/Fwe^LoseB^) marker on their surface, while the winner cells express a fit marker (Fwe^ubi^). In the less-fit cell, a closer view of the pathway involved in fitness comparison and cell death induction is shown. Interaction between Fwe^Lose^-expressing cells and Fwe^ubi^-expressing cells leads to the transcription of *azot*, which triggers the expression of the pro-apoptotic gene *hid* in the less-fit loser cell. Loser cells also secrete the protein SPARC, which downregulates *azot* expression through an unknown mechanism. Note that in this scenario, loser cell elimination occurs exclusively on contact with winner cells. Induction of apoptosis leads to loser cell delamination and cell fragmentation (blue). (D) Summary of the known isoforms in different species and their role. Dpp, Decapentaplegic; Fwe, *Drosophila* Flower protein; hFWE, human Flower protein; mFwe, mouse Flower protein; SPARC, Secreted protein, acidic, cysteine-rich.

Three main modes of cell competition have been described in *Drosophila* and mammals, during development or ageing and in disease contexts (Fig. 1): competition for limiting survival factors (Box 1; Fig. 1A) (Bowling et al., 2019; De la Cova et al., 2004; Gibson, 2005; Li and Baker, 2007; Martins et al., 2014; Parker, 2006; Vincent et al., 2011), mechanical cell competition (Fig. 1B) (Brás-Pereira and Moreno, 2018; Eisenhoffer et al., 2012; Levayer et al., 2016; Marinari et al., 2012; Norman et al., 2012; Shraiman, 2005; Wagstaff et al., 2016) and fitness fingerprints-mediated cell competition (Fig. 1C) (Coelho et al., 2018; Coelho and Moreno, 2020; Costa et al., 2020; Madan et al., 2019; Merino et al., 2013, 2016; Rhiner et al., 2010), which we discuss in detail in this Review.

In fitness fingerprints-mediated cell competition, differences in cellular fitness (Box 1) status between neighbouring cells are sensed via the expression of molecular fitness markers on their extracellular membranes. In *Drosophila*, these fingerprints are composed of three different isoforms of the conserved transmembrane protein, Flower (Fwe): Fwe^LoseA^, Fwe^LoseB^ and Fwe^ubi^ (Fig. 1D) (Merino et al., 2013). Fwe^LoseA^ and Fwe^LoseB^ isoforms are expressed in loser cells, tagging them for elimination by apoptosis through the expression of *ahuizotl* (*azot*) (Merino et al., 2016). *azot* is a single-exon gene that encodes a four EF-hand-containing cytoplasmic protein, predicted to have calcium ion-binding activity; this gene is conserved, but understudied, in multicellular animals (Merino et al., 2015). An increase in *azot* genetic dosage is associated with an extended lifespan in *Drosophila* and its deletion impairs cell competition, leading to the faster accumulation of less-fit cells, which contributes to tissue and organ ageing (Merino et al., 2015).

**Box 1. Glossary**

**Aβ42:** amyloid-β (Aβ) is a small peptide formed by the sequential cleavage of the transmembrane amyloid precursor protein (APP) by γ- and β-secretases and represents the main component of the extracellular amyloid plaques found in the brain of Alzheimer's disease patients.

**Apoptosis:** type of cell death in which a programmed sequence of events leads to the death of unneeded and abnormal cells. It is caspase dependent and results in cell fragmentation into apoptotic bodies, which are phagocytosed.

**Cell competition:** a cell fitness-sensing mechanism that occurs when cells with different fitness status are present in a tissue, leading to the elimination of those cells that, although viable, are less fit than their neighbours.

**Cellular fitness:** an as-yet unquantifiable concept referring to a quality of a cell, such as the rate of protein synthesis, used by cells to compare themselves with their neighbours. Can be affected by several factors. Cells with higher fitness are the ones surviving in heterogeneous tissues.

**Decapentaplegic (Dpp):** the fly orthologue of bone morphogenic protein, an extracellular morphogen that regulates growth and patterning.

**Entorhinal cortex layer II (ECII):** the entorhinal cortex is located between the neocortex and the hippocampus. Layer II of the entorhinal cortex (ECII) receives signals from the neocortex and sends signals to the hippocampus. The entorhinal cortex and the hippocampus are crucial brain areas for space recognition and sequence learning.

**Imaginal disc:** an imaginal disc is a sac-like epithelial structure found inside the larva that will become a portion of the outside of the adult insect during pupal transformation**.**

**Ionotropic receptor:** membrane-bound receptor that responds to ligand binding by opening an ion channel and allowing ions to flow into the cell, either increasing or decreasing the likelihood that an action potential will fire.

**Less-fit cells:** cells that are damaged but functional. Also called loser cells or suboptimal cells.

**Loser cells:** these cells are less-fit cells that are killed by their neighbours through induction of apoptosis in cell competition.

**Metabotropic receptor:** G-protein-coupled receptor. When a ligand binds to these membrane-bound receptors, the receptors activate intermediate proteins called G-proteins, which can then activate enzymes, open ion channels and initiate intracellular signalling cascades.

**Mushroom body:** a prominent bilateral structure found in the anterior regions of protostome (e.g. fruit fly) brains containing densely packed neurons. It is associated with processing olfactory sensory inputs, and olfactory discrimination and learning.

**Necroptosis:** a regulated necrotic cell death, which is caspase independent, mainly mediated by receptor-interacting protein (RIP)1, RIP3 and mixed lineage kinase domain-like (MLKL). Necroptosis serves as an alternative mode of programmed cell death.

**Scribble complex:** composed of Scrib, Dlg1 and L(2)gl and localized in the basolateral membrane. It is implicated in several signalling pathways, vesicle trafficking and the myosin II-actin cytoskeleton.

**Selective neuronal vulnerability:** selected populations of neurons are more vulnerable to damage or death in hostile conditions, whereas others are more resistant. This vulnerability can induce structural and functional alterations and might lead to neuronal death.

**Supercompetitor:** a winner, mutated cell that outcompetes wild-type cells that present a relative lower fitness, indicating an increase in its fitness over that of wild-type cells, which become losers in this scenario.

**Survival factor:** a signal that is essential for a cell to live; being deprived of such a signal would cause that cell to undergo apoptosis**.**

**Winner cell:** a cell that kills neighbouring cells that are less fit.

Here, we highlight cell competition as one possible mechanism that can promote the elimination of damaged and less-fit cells and neurons in animal models of neurodegeneration, with a focus on *Drosophila*. We also discuss the limitations of existing NDD models and the need for the development of physiologically accurate animal models. We believe that a better understanding of the pathways that promote or prevent the elimination of less-fit neurons might contribute to the development of new therapeutic targets for treating NDDs and to improve patients' quality of life.

## An overview of cell competition

Cell competition is a selection mechanism that happens throughout the life of the individual, from development to ageing and disease. Here, we present a brief historical background and discuss its high clinical potential to open new opportunities for translational research and treatments of NDDs.

As highlighted in the Introduction, cell competition is an active process by which cells are selected according to their relative fitness in a context-dependent manner, to maintain tissue and organismal homeostasis (Lima et al., 2020 preprint). Interactions between cells are thought to function as a surveillance mechanism that protects organisms from potentially dangerous cells, which could interfere with normal development, tissue function and ageing (Johnston, 2009). This mechanism has important physiological roles, such as fine-tuning the visual system during development (Merino et al., 2013), replacing old or damaged brain tissue during ageing or upon injury (Moreno et al., 2015), and protecting long-term memory (Coelho et al., 2018). In 2016, during the International Symposium on Cell competition, apoptosis and cancer in Madrid, cell competition was defined as consisting of context-dependent cell elimination via short-range, cell–cell interaction (Nagata and Igaki, 2018).

In cell competition, less-fit or damaged cells are called loser cells, whereas more-fit cells are called winner cells. Less-fit cells are viable cells that have acquired metabolic impairments, such as impaired mitochondrial function, decreased growth factors uptake or reduced protein synthesis (De La Cova et al., 2014; Morata and Ripoll, 1975; Ohsawa et al., 2012). In this context, cells with heterogeneous fitness levels emerge within a tissue as a result of mutations or external insults, and loser cells are targeted for elimination when surrounded by winner cells through several mechanisms, including apoptosis (Moreno et al., 2002a), extrusion from the epithelia (Casas-Tintó et al., 2015; Marinari et al., 2012), senescence (Bondar and Medzhitov, 2010) and phagocytosis (Li and Baker, 2007). Following loser cell elimination, tissue size increases via the compensatory proliferation of surrounding winner cells; thus, a constant tissue size is maintained (Ryoo et al., 2004). However, loser cells can remain viable in a homogeneous tissue environment in which they are in contact only with other loser cells (Tamori and Deng, 2013). This can lead to the accumulation of suboptimal cells, such as those with defects in polarity genes, that in turn can compromise the function of the tissue/organ upon their neoplastic growth (Froldi et al., 2010; Menendez et al., 2010).

**Fig. 2.**
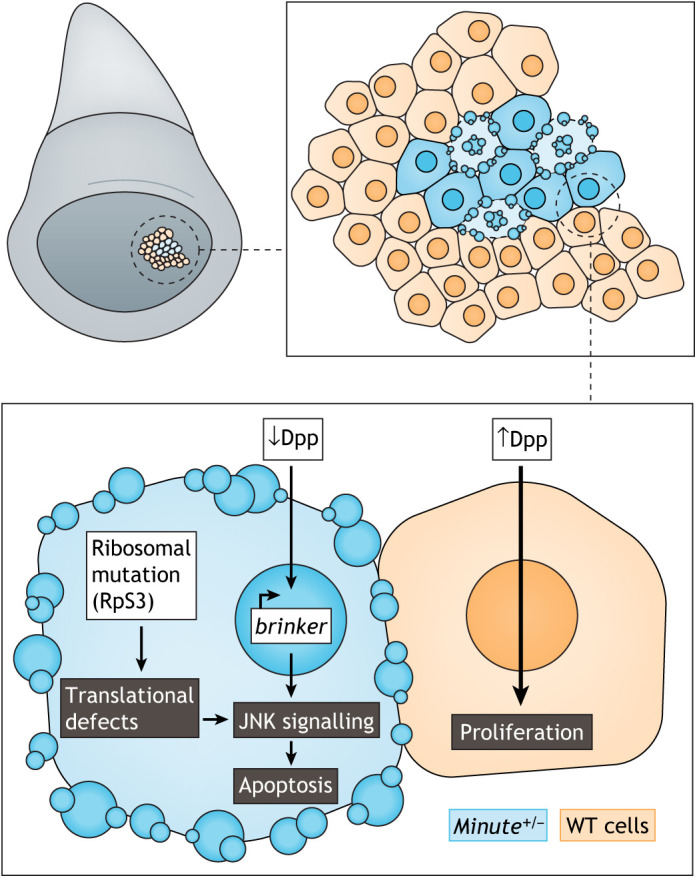
***Minute* cell competition.** (A) Loser cells (blue) surrounded by winner cells (orange) in the posterior border of the wing-pouch region in the *Drosophila* wing imaginal disc. Cells with *Minute*^+/−^ mutations are the loser cells and wild-type (WT) ones are the winners. The box contains a schematic of the mechanisms driving the loser status and leading to apoptosis of *Minute*^+/−^ cells. Decrease in Decapentaplegic (Dpp) pathway activation induces the expression of *brinker*, which in turn activates the c-Jun N-terminal kinase (JNK) pathway and promotes cell death. Additionally, proteotoxic stress is also an inductor of JNK and, consequently, apoptosis. The loser cells (blue) are thus eliminated from the tissue and the winners (orange) proliferate to compensate for the loss.

Cell competition was first discovered in *Drosophila* larvae, specifically in the wing imaginal disc (Box 1) through pioneering work from Morata and Ripoll. They described competitive interactions between wild-type cells and those lacking ribosomal genes in developing fly tissues [*Minute* (also known as *RpS17*) mutant cells] (Morata and Ripoll, 1975). Flies with heterozygous *Minute* mutations are viable and fertile, with mild phenotypic abnormalities, such as shortened bristles and slowed development (Morata and Ripoll, 1975). However, in the wing discs of *Drosophila* larvae (Fig. 2A), slowly dividing cells that contain the *Minute* mutation were progressively eliminated by apoptosis from tissue compartments containing both wild-type and mutant cells and were absent from the wings of adult flies (Morata and Ripoll, 1975).

This observation of cell non-autonomous behaviour was the first to be classed as cell competition (Morata and Ripoll, 1975), and was later confirmed by combining *Minute* mutations of varying severity (Simpson and Morata, 1981). The intensity of competition was greater for the more-severe *Minute* mutations with the slowest rate of cell division in *Drosophila*, and cell competition was shown to result from local interactions between slow- and faster-growing cells (Simpson and Morata, 1981). Interestingly, cell competition does not affect the final size of wings and compartments, indicating that wild-type cells can grow at the expense of *Minute* mutant ones (Fig. 2B). More recently, proteotoxic stress was suggested as the underlying cause of the loser status of *Minute* mutants. Baumgartner and colleagues demonstrated that cells heterozygous for the ribosomal protein RpS3 exhibit reduced autophagic and proteasomal flux and accumulate protein aggregates, whereas a rescue from competition occurs by improving their proteostasis (Baumgartner et al., 2021).

Cancer has also been linked to cell competition (Costa et al., 2020; Di Gregorio et al., 2016; Kim and Jain, 2020; Petrova et al., 2012). Fitness fingerprints-mediated cell competition has also been identified in mammals, in the context of cancer, and will be further reviewed in ‘The Flower code in cell competition’ section, below. Physiological stimuli, unrelated to genetic background, likely affect cell competition in cancer as well. For example, low-dose ionizing radiation results in p53 (also known as Trp53)-mutant cells outcompeting normal cells in the mouse oesophagus (Fernandez-Antoran et al., 2019).

Cell competition has also been described in NDDs, in a *Drosophila* model of AD. Ectopic expression of human Aβ42 (Box 1) toxic peptides decreases neuronal fitness in the fly brain, and promotes the elimination of the less-fit neurons in an Fwe-dependent manner. This neuronal elimination is beneficial for the organism as it restores motor and cognitive functions to wild-type levels (Coelho et al., 2018). More recently, our group also described that the neurons that are tagged for elimination by fitness fingerprints correspond, in part, to hyperactive neurons (Coelho and Moreno, 2020).

More research is needed to understand how fitness fingerprints-mediated cell competition induces neuronal death, to pinpoint the underlying molecules and pathways and to uncover the implications to translational research. However, cell competition is regarded as a very promising field of research to study many diseases that affect millions of people's lives. It brings a new perspective on how diseases may develop within tissues as a consequence of the accumulation or proliferation of less-fit cells and which processes fail to maintain homeostasis.

## Drivers of cell competition

Mutations in intrinsic molecular pathways and external insults trigger cell competition mechanisms. Additional factors have been implicated, including growth regulators (Martin et al., 2009) and cell polarity (Igaki et al., 2006). Although signalling and cell polarity are related to growth, the extent to which differential growth contributes to all forms of cell competition is unclear. Owing to space constraints, we will only discuss some of the better-understood triggers of cell competition.

In *Drosophila*, *Minute* mutant clones are eliminated by apoptosis in a process driven by a relative deficit in Decapentaplegic (Dpp; Box 1) pathway activation (Fig. 2). This deficit in Dpp signalling leads to the ectopic upregulation of its downstream target *brinker*, a transcriptional repressor normally inhibited by the Dpp pathway, which in turn leads to c-Jun N-terminal kinase (JNK; also known as Bsk) pathway activation and induction of apoptosis in cells with low levels of Dpp (Moreno et al., 2002a). In this system, neighbouring cells are believed to compete for the uptake of limiting survival factors, like Dpp, resulting in the elimination of less-fit cells. A similar phenomenon has also been reported in mammals: a mutation in the ribosomal protein Rpl24 leads to competitive interactions among the cells of the mouse blastocyst (Oliver, 2004).

**Fig. 3.**
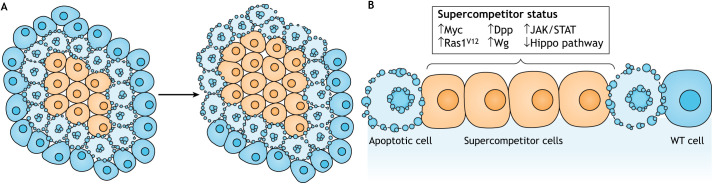
**Supercompetition.** (A) Supercompetitor cells are the winners (orange) surrounded by WT cells, which are the losers (blue). Supercompetitor cells outcompete their WT counterparts. (B) Increased levels of Myc, Ras1^V12^, Dpp, Wg and JAK/STAT signalling, as well as decreased activation of the Hippo pathway, induce the supercompetitor status of the cells. Note that Ras1^V12^ competition does not require direct contact. Dpp, Decapentaplegic; JAK/STAT, Janus kinase/signal transducer and activator of transcription; Wg, Wingless.

A fascinating discovery in the field of cell competition is supercompetition. Supercompetitor (Box 1) cells have increased fitness and can overtake a tissue by killing off their wild-type neighbours (Fig. 3). Here, supercompetitor cells are the winners and the surrounding wild-type ones are the losers (De la Cova et al., 2004; Moreno and Basler, 2004). This is analogous to cancer cells, suggesting that both cancer and supercompetitor cells may use similar mechanisms to evade normal controls on tissue growth (Baker and Li, 2008; Johnston, 2014; Moreno, 2008). Overexpression of the transcription factor Myc in *Drosophila* is sufficient to convert cells from losers into winners, outcompeting wild-type cells (De la Cova et al., 2004; Moreno and Basler, 2004).

Several mutations are now known to induce supercompetition. Many of which, like Myc, alter pathways responsible for inducing the elimination of loser cells (Bowling et al., 2019; Tyler et al., 2007). Myc is a proto-oncogenic transcription factor that targets ribosome biogenesis, including components that boost the activity of RNA polymerase I, II and III simultaneously (Campbell and White, 2014; Van Riggelen et al., 2010). Hippo pathway-mutant cells also display supercompetitive properties by taking advantage of Myc in protein biosynthesis and cellular growth to divide rapidly (Suijkerbuijk et al., 2016; Ziosi et al., 2010). The signal transducer and activator of transcription (STAT) pathway is also linked to supercompetition. The Janus kinase (JAK)/STAT pathway is a conserved signalling system that transduces cues from extracellular cytokines into transcriptional changes in the nucleus (Herrera and Bach, 2019). In flies, Unpaired (Upd) cytokines (Upd2 and Upd3) activate the receptor Domeless (Dome), which leads to the activation of JAK [also known as Hopscotch (Hop)] and STAT (also known as Stat92E) (Arbouzova and Zeidler, 2006). When STAT is absent, cells become losers and are killed by neighbouring cells, but when STAT is overactivated, cells become supercompetitors and kill surrounding cells (Rodrigues et al., 2012). Moreover, cells with overactive Wingless (Wg) signal transduction also become winners and eliminate the loser cells they surround (Giraldez and Cohen, 2003; Merino et al., 2013; Vincent et al., 2011).

The *Drosophila* Scribble complex (Box 1) has also been implicated in cell competition, specifically mechanical cell competition (Norman et al., 2012; Wagstaff et al., 2016), and new molecules that can recognize and eliminate Scribble*-*deficient cells have been identified (Yamamoto et al., 2017). Specifically, the cell surface receptor Ptp10D is recognized by the ligand Sas in wild-type cells, and both receptor and ligand relocalize to the interface between wild-type and Scribble-deficient clones (Yamamoto et al., 2017). Following this relocalization, Ptp10D is activated in loser cells, which in turn inhibits the pro-survival Egfr-Ras signalling and activates pro-apoptotic JNK signalling, leading to the death of Scribble*-*deficient cells (Yamamoto et al., 2017). *Ras* (*Ras1*; also known as *Ras85D*) is a *Drosophila* gene required for proper cell fate specification throughout development; however, a constitutively active form of Ras1 (Ras1^V12^) is sufficient to drive ectopic cell proliferation and hyperplastic tissue growth in *Drosophila* imaginal disc development (Wu et al., 2010). However, Ras1^V12^ competition does not require direct cell–cell contact (Levayer et al., 2016).

Mutations that affect the ability of a cell to compete for limited resources, such as limited survival factors and space, lead the cell to a suboptimal state and trigger competition in vertebrates and invertebrates (Merino et al., 2016). Cell competition can be considered as a two-way street: it is a surveillance mechanism that removes suboptimal cells during development and ageing to maintain the homeostasis and overall health of the organism, but it can be subverted by pre-tumoral lesions to overtake wild-type tissues and expand (Levayer et al., 2016; Merino et al., 2015). It is thus crucial to understand the fundamental genetic and molecular differences that define loser or winner cell fate.

### The Flower code in cell competition

As shown in Fig. 1, three main modes of cell competition occur in tissues. This section discusses cell competition mediated by fitness fingerprints based on the Flower code. In this cell competition mode, the internal fitness status of cells is reflected by the expression of the different isoforms of a group of cell membrane proteins encoded by the *fwe* gene (Gogna et al., 2015; Merino et al., 2015, 2013; Rhiner et al., 2010). Here, we focus on this mode of cell competition due to its newly found links to NDDs.

In *Drosophila*, the *fwe* gene encodes three protein isoforms – Fwe^ubi^, Fwe^LoseA^ and Fwe^LoseB^, as shown in Fig. 1. These transmembrane proteins differ from each other at the extracellular C-terminal domain of the protein (Rhiner et al., 2010). Fwe is also present in the membranes of synaptic vesicles and may be involved in exocytosis and endocytosis (Yao et al., 2009). These two functions are still under debate in the field.

The expression of the Fwe^LoseA^ and/or Fwe^LoseB^ isoforms is required and sufficient to label cells as losers, while the expression of Fwe^ubi^ is enough to label cells as winners (Merino et al., 2013; Petrova et al., 2012; Rhiner et al., 2010; Tweedie et al., 2009; Yao et al., 2009). These so-called fitness fingerprints are also cell and tissue specific. While Fwe^LoseA^ or Fwe^LoseB^ expression is necessary to trigger death of losers in heterogeneous epithelia of the *Drosophila* larvae wing disc, only Fwe^LoseB^ is required to eliminate neuronal cells marked as losers (Coelho et al., 2018; Merino et al., 2015, 2013; Moreno et al., 2015; Rhiner et al., 2010). Although neurons are post-mitotic cells, a role of fitness fingerprints-mediated cell selection has been shown in the adult brain upon injury and during ageing (Merino et al., 2015, 2013; Moreno et al., 2015). Our group showed that fitness-based cell selection controls the elimination of damaged tissue in *Drosophila* brain, where injury-exposed neurons induced the expression of Fwe^LoseB^ (Moreno et al., 2015). This work showed that fitness markers can be activated in adult non-proliferating tissues, like the nervous system. Moreover, previous research identified the presence of Fwe^LoseB^ and of the pro-apoptotic calcium-binding protein Azot mediating neuronal culling of incomplete or misconnected photoreceptors (Merino et al., 2013). The Flower code is cell-type specific, meaning that in the nervous system only Fwe^LoseB^ is active in loser neurons, which in turn promotes *azot* expression and apoptosis of less-fit neurons (Rhiner et al., 2010).

Fwe works downstream of many known mutations that modulate fitness in *Drosophila*, including heterozygous *Minute* mutations, supercompetition induced by Myc, and loss of polarity due to *scribble* mutations (Rhiner et al., 2010). However, the expression of Fwe^Lose^ isoforms is not induced in cells in which apoptosis is triggered by overexpression of Hemipterous, the JNK-activating kinase (Adachi-Yamada et al., 1999). The overexpression of Eiger [the fly homologue of the tumour necrosis factor (TNF) superfamily] in the eye leads to eye ablation due to massive JNK-dependent cell death, a phenotype rescued only by downregulation of Eiger or Head involution defective (Hid), but not when Fwe was downregulated (Moreno et al., 2002b; Rhiner et al., 2010). Taken together, these results suggest that Fwe is a dedicated component of cell competition-induced apoptosis and an essential mediator of fitness recognition and communication between neighbouring cells.

**Fig. 4.**
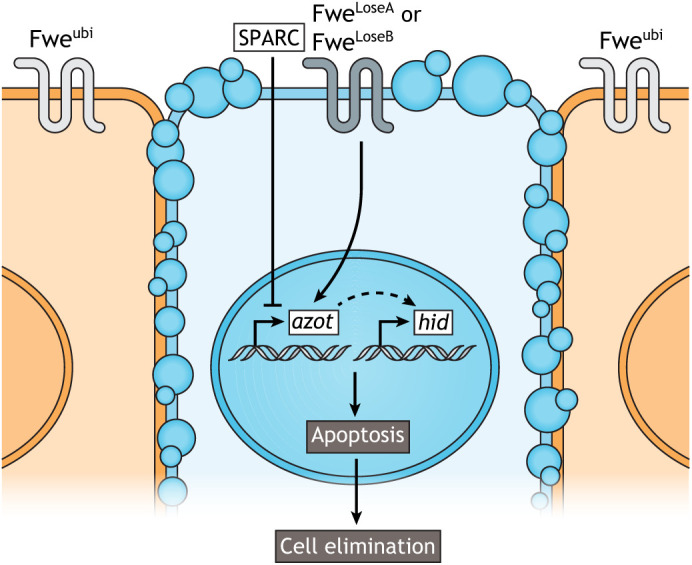
**Model of fitness fingerprints-mediated cell competition.** In a heterogeneous tissue, expression of Fwe^ubi^ in winner cells (orange) and of Fwe^LoseA/B^ in loser cells (blue) lead to the activation of *azot* in the loser cells. Azot is a fitness sensor that in turn induces the expression of the pro-apoptotic gene *hid*, leading to apoptosis and cell elimination from the tissue. SPARC counteracts the effect of Fwe^LoseA/B^ on Azot. Thus, *azot* regulation relies on the balance between Fwe status (as a readout of cell fitness) and extracellular levels of SPARC.

So how does the expression of Fwe^Lose^ lead to the elimination of loser cells? In *Drosophila*, its expression is known to promote the activation of caspase in cells expressing Fwe^LoseA^ and/or Fwe^LoseB^ (Moreno et al., 2002b; Rhiner et al., 2010). This activation is thought to trigger extrusion of the loser cell, and macrophages are recruited to clear the apoptotic debris (Casas-Tintó et al., 2015; Lolo et al., 2012). *azot* expression is also essential in this type of cell competition. Its product acts downstream of the sensing of Fwe^Lose^ and promotes apoptosis by activating the pro-apoptotic gene *hid* (Fig. 4) (Merino et al., 2016, 2015). Azot is predicted to be exclusively dedicated to cell competition-related apoptosis that integrates upstream relative fitness signals and targets loser cells for death (Casas-Tintó et al., 2015; Merino et al., 2015; Portela et al., 2010). Finally, loser elimination leads to the proliferation of winner cells to compensate for loser cell depletion to maintain the size of the organ (Moreno, 2008). However, *azot* deletion leads to the inhibition of cell competition, resulting in reduced lifespan and increased signs of tissue degeneration in fly wings and brains (Coelho et al., 2018; Merino et al., 2015). Secreted protein, acidic, cysteine-rich (SPARC), the *Drosophila* homologue of the SPARC/osteonectin protein, has an extracellular calcium-binding module (Brekken and Sage, 2000) and is a secreted extracellular matrix-associated protein involved in cell competition: prospective loser cells that express *sparc* are protected from elimination by transiently inhibiting caspase activation (Merino et al., 2015; Portela et al., 2010). The regulation of *azot* thus depends on the balance between Fwe status as a readout of cell fitness and the extracellular levels of SPARC (Fig. 4) (Martinek et al., 2008; Merino et al., 2015).

The Flower code has also been described in mice and humans. Flower proteins are known as Cacfd1 in vertebrates. The mouse *Fwe* gene (*mFwe*) encodes six different transcripts, which are typically expressed at low levels in adult tissues and translated into four protein isoforms: mFwe1, mFwe2, mFwe3 and mFwe4 (Petrova et al., 2012). Different FWE isoforms have been described in humans: hFWE1, hFWE2, hFWE3 and hFWE4 (Madan et al., 2019). In both species, these comprise two loser isoforms, mFwe1/mFwe3 and hFWE1/hFWE3, and two winner isoforms, mFwe2/mFwe4 and hFWE2/hFWE4 (Fig. 1D) (Madan et al., 2019; Petrova et al., 2012)*.* Human cell lines express four isoforms of Flower (hFWE1-4), and cells expressing hFWE2 or hFWE4 induce apoptosis of cells expressing hFWE1 or hFWE3 when co-cultured (Fig. 1D) (Madan et al., 2019). Human breast and colon cancer cells express high levels of winner hFWE isoforms, whereas neighbouring stromal cells are enriched for loser hFWE isoforms (Madan et al., 2019). Loss of winner hFWE isoforms in the cancer cells reduced tumour growth when injected into a host mouse (Madan et al., 2019). These results suggest that FWE-mediated cell competition modulates tumorigenesis in humans and, thus, further *in vivo* studies could generate new insights into FWE function.

These recent discoveries open new avenues of research in fitness fingerprints-mediated cell competition and its role in cell selection, an emerging concept in NDDs. We discuss this in the following sections.

### Neurodegenerative disorders and selective neuronal vulnerability

AD, PD and HD are among the most prevalent and best-studied diseases of the central nervous system. In this section, we briefly review the critical aspects of these NDDs that might explain why some neurons are more prone to death than others. The spick-and-span selective neuronal vulnerability (Box 1) concept is becoming important in the field, although it is still under debate. However, cell competition as a cell selection mechanism is being pointed to as a possible explanation for differences in neuronal fate (Coelho et al., 2018; Coelho and Moreno, 2020).

The susceptibility of diverse areas of the brain to age-dependent neuronal death and damage changes throughout life and across different NDDs, which reflects the diversity of symptoms identified in these diseases (Wang et al., 2010). For instance, in AD, most cell death events initially occur in neurons of the entorhinal cortex layer II (ECII; Box 1), in CA1 pyramidal cells of the hippocampus and in pyramidal neurons of neocortical association areas; later, death occurs mainly in the primary sensory cortices (Arnold et al., 1991; Hof and Morrison, 1990; Roussarie et al., 2020; West et al., 1994). In PD, neuronal loss occurs primarily in the medial part of the substantia nigra (Rinne et al., 1989). Loss of dopaminergic neurons in the substantia nigra leads to dopamine depletion in the striatum (Yang et al., 2020). In HD, the most-affected regions are the striatal caudate and putamen, the cerebral cortex and the CA1 region of the hippocampus (Reiner et al., 1988; Spargo et al., 1993; Vonsattel et al., 1985). Recent studies have reported that this loss of specific neuronal populations in NDDs is due to a selective neuronal vulnerability process (Fu et al., 2018; Roussarie et al., 2020; Surmeier et al., 2017; Wang et al., 2010).

According to the concept of selective neuronal vulnerability, specific populations of neurons are more vulnerable to injury or death under unfavourable conditions (Wang et al., 2010). Two possible types of vulnerable cells have already been established: primary cells, affected in the early stages of a disease, and secondary cells, affected later in regions where the disease has spread (Fu et al., 2018). Although this concept needs further investigation, putative explanations have started to emerge. Large pyramidal neurons belong to the most vulnerable set of neurons in AD due to their high energy requirements. These, in turn, promote high levels of oxidative phosphorylation, increasing the production of ROS, DNA and lipid modification, and, ultimately, cell death (Wang et al., 2010). The large surface area of pyramidal neurons also increases their exposure to toxic agents (Wang et al., 2010). Some of the mechanisms that might contribute to selective neuronal vulnerability include the expression of inflammatory response genes, altered synaptic transmission and synaptic vesicle transport, calcium regulation, cytoskeletal function, signal transduction and proteolytic activity (Fu et al., 2018; Wang et al., 2010). Calcium regulation also seems to be crucial for patterns of neurodegeneration. Neurons that express calcium-buffering proteins are less prone to vulnerability in AD (Hof et al., 1993), amyotrophic lateral sclerosis (Morrison et al., 1998) and HD (Morigaki and Goto, 2017).

NDDs are characterized by protein-misfolding aggregates that affect distinct brain regions, despite their widespread occurrence. In most NDDs, neuronal fate depends on the solubility of the aggregates and the efficiency of the clearance mechanism (Fu et al., 2018). Distinct interaction patterns between protein aggregates – huntingtin in HD, amyloid-β in AD, α-synuclein (α-syn) in PD – and neurons might explain the differing symptoms and neuronal stages observed in some NDDs (Babcock and Ganetzky, 2015; Roussarie et al., 2020; Sawa et al., 2003; Surmeier et al., 2017). For example, recent studies showed that, in the brains of asymptomatic PD patients, the presence of intracellular α-syn-rich protein aggregates called Lewy bodies (LBs) induced the loss of ∼10-20% of dopaminergic neurons in the ventral layer of the substantia nigra compacta; this loss was not observable in other areas affected by LBs (Dijkstra et al., 2014; Milber et al., 2012). However, in the early symptomatic stages of PD, almost all dopaminergic neurons were lost in the ventral layer, as well in other regions, in an LB-independent manner (Damier et al., 1999; Pedersen et al., 2005). Several of these vulnerable neurons affected in PD are fundamental in the neuro-modulatory control network; they are responsible for activating other neurons by neurotransmitters like dopamine and share common features like distinctive physiology with slower activity, mitochondrial stress, Ca^2+^ loading and proteotoxic stress (Dou et al., 2015; Gupta et al., 2008; James Surmeier et al., 2012; Pacelli et al., 2015; Puopolo et al., 2007; Wong and Cuervo, 2010). However, the spread of LBs in the brains of PD patients and its correlation with clinical symptoms remains poorly understood (Surmeier et al., 2017).

Moreover, each misfolded protein species and its conformation affects neuronal homeostasis networks differently, contributing to different vulnerability outcomes (Saxena and Caroni, 2011). Besides, NDD studies have revealed that protein aggregates accumulate in areas of primary vulnerability and then spread to regions of secondary vulnerability via anatomical connections (Braak et al., 2013; Braak and Del Tredici, 2017). As we discuss here, in NDDs, cellular damage accumulates, shifting neuronal health and fitness, causing different effects on neuronal fate and targeting some neurons for elimination.

Our group demonstrated that neurons could sense differences in their fitness status and compare them with the fitness levels of their neighbours in a mechanism that promotes the survival of the fittest neurons (Coelho et al., 2018; Coelho and Moreno, 2020, 2019). These results follow previous studies from the same laboratory (Merino et al., 2013), reporting that the elimination of supernumerary post-mitotic neurons during *Drosophila* eye development requires Fwe fitness fingerprints. The sensing mechanism of cell fitness through FWE might represent a general mechanism by which less-fit cells are detected and eliminated in ageing and disease contexts in a selective manner, with beneficial consequences for the organism (Bowling et al., 2019; Clavería and Torres, 2016; Coelho et al., 2018; Coelho and Moreno, 2019; De la Cova et al., 2004; Kajita and Fujita, 2015; Martins et al., 2014; Merino et al., 2016; Moreno et al., 2002a; Moreno and Rhiner, 2014; Pinal et al., 2019; Rhiner et al., 2010). However, the mechanisms that underpin the selection of less-fit/damaged neurons remain to be fully understood. In the following sections, we discuss how fitness fingerprints-based cell competition might be linked to neuronal selection in AD.

### AD models and elimination of less-fit neurons

Neuronal death is one of the hallmarks of AD, as well as accumulation of extracellular Aβ42 peptide aggregates, intracellular neurofibrillary tangles, astrogliosis, neuronal dystrophy and vascular alterations (Bedse et al., 2015; De Strooper and Karran, 2016; Selkoe, 1989; Selkoe and Hardy, 2016). The ‘amyloid cascade hypothesis’ is pointed as the leading theory explaining AD pathophysiology, proposing that insoluble Aβ plaques are the major inducers of neuronal apoptosis and neurodegeneration, as well as Tau (also known as MAPT) pathology (Akhter et al., 2014; Hardy, 2002; Oddo et al., 2003). Hence, understanding the molecular and cellular mechanisms of Aβ deposition will shed light on new treatments for AD.

Several AD mouse models have been established (reviewed in Dawson et al., 2018), recapitulating some AD phenotypes such as abundant amyloid plaques, astroglial activation, synaptic loss and dysfunction, behavioural abnormalities and neurodegeneration (Borchelt et al., 1997; Chen et al., 2000; Citron et al., 1997; Duff et al., 1996; Games et al., 1995; Hsia et al., 1999; Hsiao et al., 1996; Moran et al., 1997; Mucke et al., 2000). Nevertheless, these are still limited due to incomplete recapitulation of all disease signs. Nowadays, many animal models can simulate the initial proteinopathy, with some developing a more complete neurodegenerative cascade (Dawson et al., 2018).

To better understand the mechanisms underlying NDDs, researchers have increased their use of *Drosophila* as a model (reviewed in Jeon et al., 2020). To date, there are three major transgenic *Drosophila* AD models: the γ-secretase-based model (Guo et al., 1999; Ye and Fortini, 1999), the Tau-based models (Jackson et al., 2002; Wittmann et al., 2001), and the amyloid precursor protein (APP) or Aβ42-based models (Casas-Tintó et al., 2011; Crowther et al., 2005; Finelli et al., 2004; Jeon et al., 2020). Here, we focus on fly models based on Aβ42 expression, the most commonly used and applied in studies of cell competition. Although APP and γ-secretase are conserved in flies, the lack of β-secretase prevents APP cleavage to the toxic Aβ42. Thus, the human Aβ42 must be expressed in the fly's genome (Fossgreen et al., 1998; Luo et al., 1990; Ye and Fortini, 1998), rendering the overexpression of Aβ42 peptides in fly tissues as an artificial system. Other models, such as cells or rodents, must be employed to corroborate the findings obtained in fly models.

NDDs are complex and multifactorial, mainly due to human intricacy. *Drosophila* is an advantageous model in many respects thanks to its short life cycle combined with its abundant progeny (McGurk et al., 2015) and conservation of fundamental cellular processes (Ambegaokar et al., 2010). For instance, the nutrient-sensing pathways including the insulin/insulin-like growth factor 1 (insulin/IGF) (Berryman et al., 2008; Siddle, 2011), mechanistic target of rapamycin (mTOR) (Kapahi et al., 2004; Laplante and Sabatini, 2013), AMP kinase (Alers et al., 2012; Cantó et al., 2009) and JNK (Chimnaronk et al., 2015) pathways are conserved in *Drosophila melanogaster*, *Homo sapiens*, *Mus musculus* and *Caenorhabditis elegans* (Khan et al., 2019).

Additionally, *Drosophila* has a simpler nervous system consisting of ∼200,000 neurons compared to ∼100 billion neurons in humans. However, it is still composed of neurons and glia, protected by a blood-brain barrier, and shares organizational similarities with vertebrates (Ambegaokar et al., 2010; McGurk et al., 2015). Flies can also perform complex motor behaviours and are amenable to experimental memory and learning assays (Coelho et al., 2018). Together, these traits make *Drosophila* a valuable model organism to study NDDs and perform genetic and pharmacological screens. Because not all models can simultaneously mimic all disease phenotypes/symptoms, choosing a model will affect the results and needs to be done with regard to phenotypes and scope of the experiment.

As explained above, in NDDs, neurons are not affected equally or simultaneously, which indicates some selectivity. In this section, we discuss how cell competition is involved in the elimination of the less-fit neurons in AD, explaining how some neurons are more prone to elimination than others, which will eventually help to understand the diversity of symptoms observed in NDDs. This is a new field and the results from our own work were achieved by using a *Drosophila* AD model developed by Sergio Casas-Tintó and colleagues (Casas-Tintó et al., 2011). This model was generated by expressing two copies of the human Aβ1-42 (hAβ42) fused to a secretion signal peptide under the control of the upstream activating sequence (UAS) (Fernandez-Funez et al., 2007). Overexpression of the two copies of hAβ42 mimics the APP duplication linked to early-onset familial AD (Rovelet-Lecrux et al., 2006) and drives more robust phenotypes in the *Drosophila* eye. This can be critical for identifying the mechanisms involved in Aβ-induced neuronal toxicity and would not be possible with a knock-in model (Casas-Tintó et al., 2011). These flies show small and disorganized/unpatterned eyes with necrotic spots and thin and disorganized retinas with poorly differentiated photoreceptors (Casas-Tintó et al., 2011).

Apoptosis is the main pathway of neuronal death in AD (Roth, 2001). Ectopic expression of human Aβ42 in the fly AD model was reported to induce neuronal apoptosis, locomotive dysfunction and decreased lifespan, recapitulating traits seen in patients (Hong et al., 2011). Studies using the same *Drosophila* AD model revealed that Aβ42-induced neurotoxicity can be triggered early in fly development, and inhibiting caspases by expressing the baculovirus P35 protein rescued Aβ42-induced cell death in eye imaginal discs (Moreno and Basler, 2004). In adult flies, inhibiting caspases only partially rescued the small and disorganized Aβ42-eye phenotype (Tare et al., 2011). These results implicate caspase-mediated cell death in Aβ42-linked neurotoxicity in the developing *Drosophila* eye and suggest the involvement of other caspase-independent compensatory pathways in adult flies (Tare et al., 2011). According to previous studies, inhibition of the JNK-mediated apoptotic pathway can also protect murine neurons from death in an AD context (Braithwaite et al., 2010). Tare and colleagues also found that Aβ42 activates JNK signalling in the adult *Drosophila* eye. JNK and caspase inhibition decreased cell death, resulting in flies with large and well-developed eyes, with a total rescue of the Aβ42-overexpression phenotype (Tare et al., 2011). The involvement of several pathways in Aβ42 neurotoxicity needs to be considered when studying the development of new therapies. Hong et al. and Tare et al. performed their studies using a model developed by Finnelli and colleagues, expressing human Aβ42 sequence in *Drosophila* nervous system tissues (Finelli et al., 2004). We should clarify that the severity of Aβ42 is age and dose dependent (Finelli et al., 2004). Furthermore, in the same AD model developed by Finelli et al., pharmacological inhibition of the JNK/Forkhead box O (Foxo) signalling pathway rescued neuronal cell death in the brain and eyes, as well as rescued the reduced survival rate and locomotor impairments (Hong et al., 2012). These findings indicate that Aβ42 induces neurotoxicity in *Drosophila* through JNK and Foxo activation (Hong et al., 2012). Foxo is a transcription factor that induces the pro-apoptotic gene *hid*, via JNK signalling, and it has been associated with NDDs (Hong et al., 2009; Hu et al., 2019).

The role of different Aβ42-induced pathology in the aetiology of AD is under intensive investigation. Post-mortem studies have revealed that a range of morphological and biochemical changes occur in AD patients' brains, including apoptosis, as evidenced by the occurrence of DNA fragmentation and autophagic vacuoles (Mukhin et al., 2017; Smale et al., 1995; Yamatsuji et al., 1996). Also, insulin-deficient signalling promoting neuronal death has been described in AD (Chen et al., 2014; Erol, 2009; Guo et al., 2017). Recently, drugs used to treat type 2 diabetes showed a surprising neuroprotective effect in a mouse model of AD (Tai et al., 2018). Besides disease progression, brain tissue of AD patients showed JNK and FOXO activation, both promoting neuronal death (Cole et al., 2007; Zhu et al., 2001). Furthermore, brains of AD patients display high amounts of ROS, which have been reported to increase Aβ42 levels and promote its accumulation (Misonou et al., 2000). Mitochondria are essential to neurons due to their high energy requirements (Jeanneteau and Arango-Lievano, 2016). Consequently, mitochondrial deficits have also been associated with several NDDs (Gan et al., 2018). Impairments in mitochondrial fission and biogenesis, defective mitochondrial trafficking, high proteotoxic stress and decreased glucose transportation lead to a hypometabolic state in the brain and to a decrease in glucose, features used to stage AD progression (Correia et al., 2016; DuBoff et al., 2013; Sorrentino et al., 2017).

Because impaired proteostasis is associated with ageing and age-related pathologies, tissue homeostasis and longevity may rely on the evaluation of cell fitness (Kaushik and Cuervo, 2015; Taylor and Dillin, 2011). To the best of our knowledge, we are the only group working in neuronal selection based on cell competition in the context of AD. However, as discussed above, Baumgartner and colleagues also investigated proteotoxic stress as the cause of loser status in a Machado–Joseph's disease *Drosophila* model expressing the human aggregate-prone polyQ protein ataxin-3 (SCA3/MJDQ78; also known as ATXN3) (Baumgartner et al., 2021; Bonini, 1999). Patches of cells overexpressing SCA3/MJDQ78 in the wing imaginal disc, in wild-type background, showed increased apoptosis at the borders and decreased growth rates, mimicking the loser status in *Minute* competition (Fig. 2) (Baumgartner et al., 2021). These results could suggest that proteotoxic stress, a common feature in NDDs, has a role in determining the fitness of neurons and could help to explain neuronal death. How exactly proteotoxic stress induces the loser status remains to be understood. Genes involved in energy metabolism, proteotoxic and oxidative stress responses, and protein metabolism promote cell competition, suggesting that neurons with a deficient energy supply or metabolism might be more prone to being eliminated by neuronal selection induced by their fitness status.

**Fig. 5.**
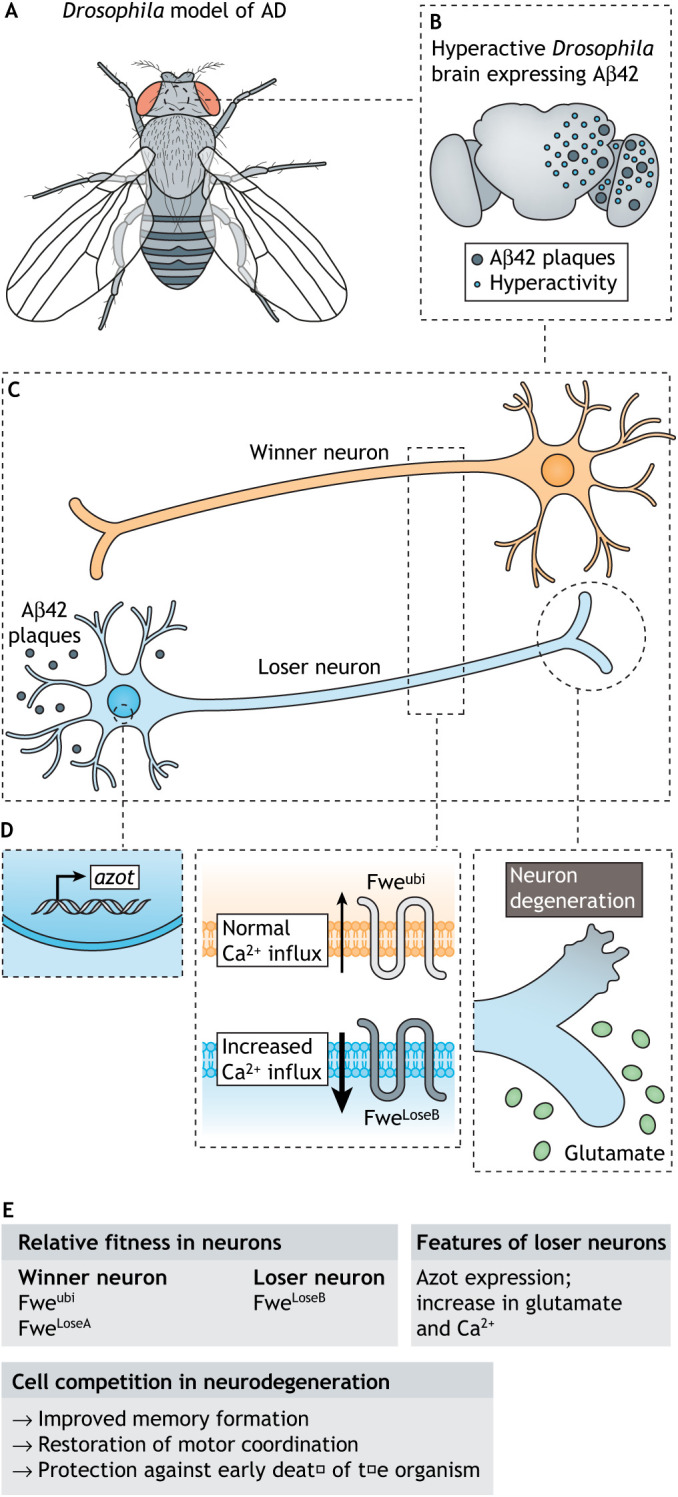
**Cell competition and Alzheimer's disease (AD).** (A-C) In a *Drosophila* model of AD (A), ectopic expression of Aβ42 in the brain (B) is harmful to neurons and decreases their fitness (C). (D) These differences in the relative fitness are sensed by Fwe isoforms through the Flower code, with loser (blue) neurons expressing Fwe^LoseB^ and winner (orange) neurons expressing Fwe^ubi/LoseA^. Expression of *azot* leads to apoptosis and degeneration of the loser neuron; moreover, the loser neuron increases Ca^2+^ influx and in hyperactivity due to higher concentrations of glutamate. (E) Summary of how the removal of dysfunctional neurons is beneficial to AD. Winner neurons expressing Fwe^ubi/LoseA^ promote the death of the hyperactive loser neuron, which expresses Fwe^LoseB^ and Azot. This mechanism improves memory formation and re-establishes motor coordination, protecting against early death and brain ageing in *Drosophila*, a mechanism that may also occur in mammals.

The first link between cell competition and neurodegenerative disease was found using the above-mentioned Casas-Tintó et al. *Drosophila* model of AD (Fig. 5A,B) (Casas-Tintó et al., 2011; Coelho et al., 2018; Coelho and Moreno, 2020). Our group showed that accumulation of human Aβ42 induces damaged suboptimal neurons (Fig. 5C) that upregulate the expression of the Fwe^LoseB^ isoform and Azot. Loser Aβ42-damaged neurons underwent apoptotic cell death, as measured by an increase in the levels of the caspase DCP1 (Fig. 5D) (Coelho et al., 2018). Aβ42-induced cell death was detected in an autonomous and non-autonomous manner in the neurons of the *Drosophila* eye imaginal discs. In this model, cells expressing Aβ42 behaved as losers and were eliminated over time from the neuronal epithelium in a fitness fingerprints-dependent manner. The removal of neurons through cell competition revealed a keen beneficial effect for the organism, protecting against motor decline, memory impairments and brain degeneration (Fig. 5E) (Coelho et al., 2018). The same study reported that ectopic expression of a pathogenic form of the human huntingtin gene (HTT-Q128), which carries an extended glutamine tract found in HD patients, induced low neuronal fitness, but the same was not observed upon expression of a Parkinson-related human α-synuclein A30P mutation (Coelho et al., 2018). These observations imply that low neuronal fitness is not a common feature across NDDs. Rather, it is associated with stages of neurodegeneration and depends on the toxicity elicited by the protein aggregates, thus being disease-specific.

As in post-mortem AD patient brains, Coelho and colleagues show that the brains of adult AD flies expressing hAβ42 oligomers develop abnormal brain morphology, particularly an increased incidence of neurodegenerative vacuoles. Azot knockdown not only exacerbated the brain vacuoles but also further shortened the lifespan of these flies (Coelho et al., 2018; Jellinger and Stadelmann, 2001). Conversely, introduction of one extra functional copy of *azot* in the Aβ42 *Drosophila* model of AD, which stimulated the elimination of less-fit neurons, was enough to rescue brain morphology and to ameliorate motor coordination and long-term memory formation (Coelho et al., 2018). These results are in accordance with the previously discussed notion that fitness fingerprints-mediated cell selection occurs during physiological brain ageing and promotes the increase in the fly lifespan (Merino et al., 2015) (see ‘The Flower code in cell competition’ section). *azot* is required for tissue health maintenance in adulthood. When *Drosophila* brain tissues exhibit less-fit cells, the absence of *azot* increases morphological malformations and accelerates age-dependent degeneration (Merino et al., 2015). By contrast, the expression of one additional copy of *azot* is beneficial and increases lifespan in flies (Merino et al., 2015). Together with the results described by Coelho et al., we show that this fitness fingerprints-mediated cell selection can be a common signal of neuronal selection, not only in the context of NDDs but also upon brain injury or ageing (Coelho et al., 2018; Merino et al., 2013, 2015; Moreno et al., 2015).

In conclusion, these findings indicate, for the first time, that misfolding-prone toxic Aβ42 affects neuronal fitness, and that induction of fitness-based apoptosis is beneficial and protective in a fly model of AD (Coelho et al., 2018). Similarly, neuronal fitness is reduced in an HD *Drosophila* model expressing a pathogenic form of huntingtin (Coelho et al., 2018). Targeting key apoptotic players to physiologically increase apoptosis may be a new approach to treat AD, improving the symptoms of early symptomatic patients by eliminating dysfunctional neurons. Thus, further studies in cellular or mouse NDD models are needed to complement these findings. However, as fitness fingerprints-mediated cell competition is conserved in flies, mice and humans (Madan et al., 2019; Merino et al., 2013; Petrova et al., 2012; Rhiner et al., 2010), it is appealing to expect similar results in other models.

Knowing that the cell competition cascade is induced in dysfunctional neurons in several contexts, the next section will address neuronal hyperactivation, a key feature of damaged neurons, and how it may induce cell competition.

### Hyperactivity-induced fitness fingerprints

Throughout adult life, organisms accumulate damaged but viable neurons leading to a dysfunctional neuronal network and imbalance between stability and plasticity (Frere and Slutsky, 2018). Impaired activity of hippocampal and cortical circuits, as well as dysfunctions in synaptic and neuronal plasticity, have been extensively studied in AD (Haberman et al., 2017; Mesulam, 1999; Mucke and Selkoe, 2012; Palop and Mucke, 2010). The urgent need to identify effective therapies able to prevent, halt or reverse AD reveals the importance of finding new approaches and considering other factors beyond Aβ42. For instance, a growing body of evidence supports neuronal hyperactivity as a major player in the progression of AD (Palop and Mucke, 2009). Hyperactive neurons disrupt healthy neuronal function and are harmful for neural communication, contributing to learning and memory impairments (Bakker et al., 2012, 2015; Sanchez et al., 2012). This section will review how neuronal cell competition and hyperactive neurons are related, and how studying hyperactivity in AD can be a new approach to developing therapies.

The main excitatory neurotransmitter in the central nervous system is glutamate, which is essential for memory, neuronal development and synaptic plasticity (Ribeiro et al., 2017). Glutamate receptors that are calcium permeable can be ionotropic (Box 1), promoting rapid excitatory neurotransmission, including via the N-methyl-d-aspartate (NMDA), α-amino-3-hydroxy-5-methyl-4-isoxazolepropionic acid (AMPA), and kainate receptors (Mayer, 2005). These receptors can also be metabotropic [Box 1; metabotropic glutamate receptors (mGluRs)], inducing a signalling transduction cascade in the cytoplasm upon a prolonged stimulus (Gerber et al., 2007). Their involvement in pre-synaptic and post-synaptic processes potentiates the fine-tuning of calcium signalling (Verma et al., 2018). Overstimulation of calcium signalling has been implicated in AD, PD and HD, with calcium dysregulation also playing a pathogenic role (Calabresi et al., 1999; DeLong and Wichmann, 2015; Hsieh et al., 2006; Shankar et al., 2007).

Calcium from the synaptic activity is stored in the mitochondria and endoplasmic reticulum of dendritic spines, enabling neuronal recovery (Koch et al., 1992). Evidence from post-mortem studies of AD, PD and amyotrophic lateral sclerosis has revealed that neurons show dendritic spine loss and shortening and simplification of dendritic arbors, as well as mitochondrial depletion, and these dysfunctional neurons are possibly more prone to cell death (Baloyannis et al., 2004; Cherra et al., 2013; Dagda et al., 2014; Hammer et al., 1979; Stephens et al., 2005). Cytoplasmic calcium overload is considered a neuronal trigger that induces cell death in AD models (Kuchibhotla et al., 2008; Supnet and Bezprozvanny, 2010), and the excessive stimulation of NMDA and other receptors by deregulated synaptic glutamate has been connected to cell death caused by apoptosis and necroptosis (Box 1) in NDDs (Dong et al., 2009; Esposito et al., 2013; Selimi et al., 2000; Zuo et al., 1997).

Neuronal hyperactivity has also been detected in the early stages of AD in the cortex and hippocampus of AD patients and mouse models, being less evident in more-advanced disease stages (Busche et al., 2012). Increased neuronal hyperactivity, together with Aβ42 expression and morphological changes, may reduce the fitness of neurons, culminating in eliminating the less-fit neurons. Therefore, our group decided to investigate the role of fitness fingerprints-mediated cell competition in AD-related hyperactive neurons (Coelho and Moreno, 2020). First, using the same genetic construct developed by Sergio Casas-Tintó and colleagues to model AD in *Drosophila* (Casas-Tintó et al., 2011), the team confirmed that ectopic expression of human Aβ42 could induce neuronal hyperactivity in several *Drosophila* brain regions through calcium overloading and increased levels of glutamate. These hyperactive neurons induced the expression of Fwe^LoseB^ in the axonal membrane and the expression of the fitness sensor Azot, leading to cell selection and neuronal death (Fig. 5).

Yao et al. proposed that Flower^LoseA^ works as an essential calcium channel for endocytosis of synaptic vesicles in neuromuscular junctions (Yao et al., 2009). However, further evidence is scarce and the topic is still under discussion (Chang et al., 2018; Madan et al., 2019; Xue et al., 2012). The calcium-channel hypothesis was also assessed in a study by Coelho and Moreno, which confirmed that expression of mutant Fwe^LoseA^ without Ca^2+^ channel capacity is enough to mark clones for elimination through fitness comparison, independently of a potential Ca^2+^ influx (Coelho and Moreno, 2020), refuting the idea of Fwe^LoseA^ working primarily as a calcium channel. In the same study, RNA interference-mediated knockdown of both Fwe^Lose^ isoforms reduced Aβ42-induced apoptosis of hyperactive neurons in the mushroom body (Box 1). The authors also evaluated whether artificial silencing of these hyperactive neurons this would be enough to improve Aβ42-induced neuronal death and could be used as a potential therapeutic mechanism. As expected, artificial neuronal silencing by promoting neuronal hyperpolarization with Kir2.1 (an inward-rectifier K^+^ channel) reduced the levels of Fwe^LoseB^ in an Aβ42-induced hyperactivity context (Coelho and Moreno, 2020).

This section reviewed neuronal hyperactivity and how Aβ42 promotes selection based on fitness fingerprints-mediated cell competition, marking and removing these highly active neurons. Growing evidence has pointed to neuronal hyperactivity as an emergent hallmark of AD (Frere and Slutsky, 2018; Lerdkrai et al., 2018). Treatments with anti-epileptic drugs have improved cognitive performance upon rescuing network and synaptic abnormalities in the hippocampus of mouse models of AD and in patients at risk of developing AD (Bakker et al., 2015; Sanchez et al., 2012). Accordingly, decreasing neuronal hyperactivity could modulate disease progression. As discussed before, this study was conducted only in *Drosophila* and needs further genetic and pharmacological validation in mammalian systems. Also, the exact role of the fitness fingerprints machinery in the human brain is still unknown. Besides, the relatively simple *Drosophila* brain anatomy lacks some structures and circuits present in the human brain, which prevents a complete assessment of a multifactorial approach to AD. Nevertheless, we believe that the findings discussed above help shed light on the complexity of AD and open new avenues for therapeutic targets.

### Conclusions and future perspectives

The prevalence of NDDs such as AD or PD is increasing worldwide due to the increasing life expectancy. Despite years of research in AD, drug development and clinical trials targeting Aβ42 or Tau have failed, probably due to the multifactorial nature of AD (Bettens et al., 2013; Jeon et al., 2020; Mehta et al., 2017). Understanding the molecular mechanisms regulating the fitness status of neurons in several contexts might give us insights into how the selective loss of neuronal populations occurs in neurodegeneration and the role of cell competition in eliminating the suboptimal but still viable neurons. Studies discussed in this Review, although few, have implicated cell competition in the modulation of AD progression, supporting a new alternative perspective on the mechanisms that contribute to neurodegeneration (Coelho et al., 2018; Coelho and Moreno, 2020, 2019). We believe that, in the future, the field needs to uncover the molecular mechanisms upstream and downstream of Fwe and Azot, learn how cells sense the fitness status of their neighbours, and investigate which are the hypothetical membrane receptors or secreted molecules essential for this communication. Addressing these questions in different contexts, like in NDDs, might help understand why a broad expression of Aβ42 predisposes some neurons to damage and death while others remain healthy. This Review tackled signalling pathways and cellular modifications that lead to neuronal selection and death. We think that identifying the major signalling event responsible for massive neurodegeneration could provide a clue for therapeutic intervention in several NDDs. Conservation of the fitness fingerprints machinery in flies (Coelho et al., 2018; Rhiner et al., 2010), mice (Petrova et al., 2012) and humans (Madan et al., 2019) justifies further studies across models to help the field understand how cell competition modulation may be therapeutically used in NDDs, improving clinical symptoms.
